# Current Challenges in the Identification of Pre-Erythrocytic Malaria Vaccine Candidate Antigens

**DOI:** 10.3389/fimmu.2020.00190

**Published:** 2020-02-21

**Authors:** Paulo Bettencourt

**Affiliations:** The Jenner Institute, University of Oxford, Oxford, United Kingdom

**Keywords:** *Plasmodium falciparum*, pre-erythrocytic malaria, liver-stage, infectivity, vaccine design, antigen identification, immunopeptidomics

## Abstract

*Plasmodium* spp.-infected mosquitos inject sporozoites into the skin of a mammalian host during a blood meal. These enter the host's circulatory system and establish an infection in the liver. After a silent metamorphosis, merozoites invade the blood leading to the symptomatic and transmissible stages of malaria. The silent pre-erythrocytic malaria stage represents a bottleneck in the disease which is ideal to block progression to clinical malaria, through chemotherapeutic and immunoprophylactic interventions. RTS,S/AS01, the only malaria vaccine close to licensure, although with poor efficacy, blocks the sporozoite invasion mainly through the action of antibodies against the CSP protein, a major component of the pellicle of the sporozoite. Strikingly, sterile protection against malaria can be obtained through immunization with radiation-attenuated sporozoites, genetically attenuated sporozoites or through chemoprophylaxis with infectious sporozoites in animals and humans, but the deployability of sporozoite-based live vaccines pose tremendous challenges. The protection induced by sporozoites occurs in the pre-erythrocytic stages and is mediated mainly by antibodies against the sporozoite and CD8^+^ T cells against peptides presented by MHC class I molecules in infected hepatocytes. Thus, the identification of malaria antigens expressed in the sporozoite and liver-stage may provide new vaccine candidates to be included, alone or in combination, as recombinant protein-based, virus-like particles or sub-unit virally-vectored vaccines. Here I review the efforts being made to identify *Plasmodium falciparum* antigens expressed during liver-stage with focus on the development of parasite, hepatocyte, mouse models, and resulting rate of infection in order to identify new vaccine candidates and to improve the efficacy of the current vaccines. Finally, I propose new approaches for the identification of liver-stage antigens based on immunopeptidomics.

## Introduction

According to the latest WHO report, around 219 million clinical episodes of malaria were reported in 91 countries, most of these occurring in sub-Saharan African countries, representing a decrease of 1 million cases over the previous year. 435,000 deaths were registered in 2017 due to malaria, identical to the numbers of reported deaths in 2015. Dramatically, most of these deaths are African children under 5 years of age (1). Increasing parasite drug resistance and mosquito insecticide resistance threatens to lead to worse control and higher mortality in the coming years (2). Therefore, the control and eventual eradication of this disease relies on the development of a highly effective malaria vaccine. The vaccine RTS,S/AS01, the first ever malaria vaccine to enter Phase III clinical trials and the first human parasitic vaccine ever created, shows modest efficacy, short durability and needs to be administrated in a four-dose schedule for maximum efficacy (3). Nevertheless, RTS,S/AS01 brings hope for the development of more efficacious vaccines. Currently there are about 20 vaccines in clinical trials based on whole organisms or very well-known antigens, reviewed elsewhere (4, 5). The Malaria Vaccine Technology Roadmap proposes two main objectives for the development of new malaria vaccines by 2030: (1) vaccines with protective efficacy of at least 75% against clinical malaria and, (2) vaccines that reduce transmission of the parasite to reduce the incidence of human malaria infection (6). Vaccination with the pre-erythrocytic malaria vaccine RTS,S induces high levels of antibodies (7), CD4^+^ T cells (8, 9), and CD8^+^ T cells (9) specific for the circumsporozoite protein (CSP). To improve the efficacy of pre-erythrocytic malaria vaccines, however, there is need to identify new vaccine candidate antigens, especially antigens able to induce strong CD8^+^ T cell responses.

It has been 50 years since Ruth Nussenzweig's seminal paper describing that radiation-attenuated sporozoites induce sterile protection (10), remain in the liver (11) and require CD8^+^ T-cell responses for protection (12) against the CSP protein (12), and a single epitope, known as Pb9, can induce protection in mice (13, 14). In the 1990's, our group has developed a CSP *P. berghei* vaccine that can induce sterile protection in mice, based in a Modified Vaccinia Virus viral vector (13). Protection can be induced with peptides presented by MHC Class I molecules in the mouse liver, mediated by CD8^+^ T cells, using viral vectors against a single antigen (13). In humans, there is an important role of CD8^+^ T cells in inducing protection against pre-erythrocytic malaria. It has been shown in naturally exposed individuals or volunteers vaccinated with radiation-attenuated sporozoites (RAS) that these responses are against CSP and other antigens, which have not been extensively characterized (15, 16). Moreover, it has been shown in clinical trials with ChAd63-MVA expressing ME-TRAP that CD8^+^ T cells correlate with protection (17). Thus, the identification of antigens presented by MHC-I molecules in infected hepatocytes may provide the yet unidentified antigens required for full protection. This review focuses on the work that has been done to identify liver-stage protective antigens from *P. falciparum*, and proposes new strategies to complement or improve current methods of antigen discovery for malaria vaccines.

### Biology of *Plasmodium*

*Plasmodium* sp. are polymorphic, obligate intracellular parasites with a complex life cycle that has both an asexual and a sexual stage and parasitize two very different hosts: an invertebrate host, mainly *Anopheles* mosquitos, and a vertebrate mammalian host. Five species *P. falciparum, P. vivax, P. ovale, P. malariae*, and *P. knowlesi* are recognized as responsible of natural infection in humans, although infections in other primate species have been reported with these pathogens (18).

Malaria is transmitted by different species of *Plasmodium*-infected *Anopheles* female mosquitos (18). The infected female mosquito injects approximately one hundred sporozoites into the skin of a mammalian host whilst taking a blood meal (19). The sporozoites search for capillaries in the skin and, within minutes, enter the host's circulatory system, eventually infecting cells in the liver (20, 21). In the liver, the sporozoites traverse several hepatocytes before establishing a successful infection within a hepatocyte (22, 23), residing in a parasitophorous vacuole with specialized functions (22, 24). The hepatic infection is asymptomatic and takes about 7 days to complete for *P. falciparum*. *P. vivax* and *P. ovale* can establish a latent form, the hypnozoite, that persists in the liver and may cause relapses by invading the bloodstream months or years later (18). In the hepatocytes, parasites undergo asexual schizogony to form tens of thousands of merozoites (18). The parasites inside the hepatocyte are known as exo-erythrocytic forms (EEFs). The pre-erythrocytic stage or exo-erythrocytic cycle includes both the sporozoite invasion of the mammalian host and the liver-stage. This stage represents a bottleneck in the disease which is ideal to block progression to clinical malaria, through chemotherapeutic and immunoprophylactic interventions.

Following merozoite egress from the infected liver cell, the parasites escape into the blood circulation to infect erythrocytes, where they asexually replicate exponentially. This constitutes the symptomatic and well-studied blood-stage malaria or erythrocytic cycle (18). Once merozoites invade erythrocytes, they become trophozoites that develop into schizonts which eventually rupture the host cell and re-infect new erythrocytes. Alternatively, some trophozoites develop into gametocytes. If a female mosquito takes a blood meal from an infected mammalian host, containing at least one female and one male gametocyte, sexual development of the parasite resumes (18). Inside the mosquito, the sexual stage or sporogonic cycle occurs, forming gametes, which develop into ookinetes that cross the mosquitoes' mid gut wall to become oocysts. Inside oocysts, thousands of sporozoites are formed that eventually migrate to the salivary glands (18, 25).

### Major Achievements Leading to the Identification of Pre-Erythrocytic Proteins

#### Antigen Identification Based on Genomic, Transcriptomic, and Proteomic Studies

In 2002, *P. falciparum* genome was sequenced, which constituted a landmark in the field and the beginning of the post-genomic era. *P. falciparum* nuclear genome consists of an unusually high A-T content, 22.8 megabases long, composed of 14 chromosomes encoding 5,268 genes (26). Surprisingly, only 733 (14% of total) genes encoding enzymes and transporters were identified but a large proportion of genes were thought to be involved in host–parasite interactions and immune evasion (26). Also, many proteins seem to be targeted to the apicoplast, which is an organelle homologous to the chloroplasts of plants and algae, with a role in the anabolism of fatty acids, isoprenoids and haeme. The apicoplast is present in many organisms from the phylum apicomplexa and is essential for parasite survival (26). In the same year, the first large-scale, high-throughput proteomics study on four stages of the parasite (sporozoites, merozoites, trophozoites and gametocytes) was published revealing 2,415 proteins in total and 1,049 in the sporozoite but the liver stage was not surveyed (27) (Table 1). Interestingly, the antigenically variant proteins (var and rif) known to be expressed on the surface of infected erythrocytes were also detected in sporozoites (27), which are thought to be involved in immune escape and suggests a promiscuity in gene expression between stages. Moreover, chromosomal clusters of proteins expressed simultaneously were also identified (27), suggesting some form of gene expression regulation. Both studies were performed with *P. falciparum* clone 3D7. In a back-to-back publication, another large-scale proteomics study revealed 1,289 proteins in three stages of the parasite (asexual blood stages, gametocytes and gametes), using *P. falciparum* isolate NF54 (34) (Table 1). The pre-erythrocytic stages were not included in this study. The combined protein identifications of these two studies represent 52% of the predicted gene products (2,731) (26), and in both studies infected hepatocytes were not included. The genome sequencing and the first two large-scale proteomics studies brought new hope for the identification of new targets for therapies as well as new vaccine candidates. Regrettably, liver-stage proteins were not identified, thus urging for the identification of antigens expressed at this stage. However, in an integrated study that combined both transcriptome and proteome analysis, Kappe produced *P. yoelii* expressing GFP which allowed the isolation of liver-stage-infected hepatocytes, through cell sorting. This allowed performing gene and protein expression directly in purified infected hepatocytes, which resulted in the identification of 1,985 genes expressed during liver-stage using *P. yoelii* microarrays. This revealed interesting genes such as the fatty acid synthesis II (FASII) and other apicoplast pathways as potential druggable targets for malaria prophylaxis (38).

**Table 1:** Major achievements leading to the identification of pre-erythrocytic proteins in chronological order.

**Year**	**Achievement**	**Discovery strategy**	**Description/Method**	**Number of proteins identified**	**Organism**	**Stage**	**References**
**PRE-GENOMIC ERA**							
1980	Circumsporozoite protein identification	Immuno-fluorescence assay (IFA)	Hybridoma generated by the fusion of myeloma cells with splenocytes from mice immunized with *P. berghei*	1	*P. berghei*	Sporozoite	(28)
1987	LSA-1 identification	IFA and ELISA	Screening of serum reactive against pre-erythrocytic stages	1	*P. falciparum* isolate Tak 9.96	Liver-stage	(29)
1988	TRAP identification	Gene cloning and sequencing	Gene cloning and sequencing of TRAP	1	*P. falciparum* isolate Tak 9.96	Sporozoite	(30)
1994	STARP Identification	IFA	Gene cloning and sequencing of STARP	1	*P. falciparum*	Sporozoite, liver-stage	(31)
1996	SALSA Identification	IFA and ELISA	Gene cloning and sequencing of SALSA	1	*P. falciparum* isolate NF54	Sporozoite, liver-stage	(32)
2000	LSA-3 Identification	Screening of clones	Gene cloning and sequencing of LSA-3	1	*P. falciparum* K1 strain	Sporozoite, liver-stage	(33)
**POST-GENOMIC ERA**							
2002	*P. falciparum* genome sequencing	Shotgun sequencing	*P. falciparum* genome sequence	5,268 predicted proteins	*P. falciparum* clone 3D7	N/A	(26)
2002	First *P. falciparum* 3D7 proteome sequencing	Proteomics	High-throughput liquid chromatography and tandem mass spectrometry	2,415 in total,1,049 in the sporozoite	*P. falciparum* clone 3D7	Sporozoites, merozoites, trophozoites and gametocytes	(27)
2002	First *P. falciparum* NF54 proteome sequencing	Proteomics	Proteomics using nano LC-MS/MS	1,289	*P. falciparum* isolate NF54	Trophozoites/schizonts, gametocytes and gametes	(34)
2003	First screen to identify pre-erythrocytic antigens	Bioinformatics	Bioinformatics predictions, HLA-supertype considerations *in vitro* cellular assays assays to identify potentially immunogenic antigens	16	*P. falciparum*	Sporozoite and liver-stage	(16)
2004	First screen to identify sporozoite proteins	Transcriptomics	Transcriptomics based on suppression subtractive hybridization (SSH)	25	*P. yoelii* strain 17XNL	Sporozoite compared to merozoite	(35)
2004	First transcriptomic study using EEF axenic culture	Transcriptomics	Generation of expressed sequence tags (ESTs) from axenic culture of EEFs	652	*P. yoelii* strain 17XNL	EEFs	(36)
2005	First transcriptomic study using laser microdissection of liver-stage forms	Transcriptomics	Laser capture microdissection and generation of ESTs	623	*P. yoelii* strain 17XNL	Liver-stage	(37)
2008	First liver-stage combined transcriptome and proteome	Transcriptomics and proteomics	Microarray transcriptomic and 1D LC-MS/MS shotgun proteomic analysis of murine liver-stage	1,985 transcripts and 712 proteins	*P. yoelii* expressing GFP and strain 17XNL	Liver-stage	(38)
2008	First transcriptomic study to look at the transition from the insect to the mammalian host	Transcriptomics	Transcriptomics of salivary gland sporozoites vs. sporozoites co-cultured with hepatocytes based on *P. falciparum* microarrays	532	*P. falciparum* isolate NF54	Sporozoite transition to liver-stage	(39)
2010	Transcriptomic focused on the development of liver-stages	Transcriptomics	Transcriptomics of wild type and RAS and liver-stages during development	1,133 genes	*P. yoelii* strain 17XNL	Sporozoite and liver-stage	(40)
2011	First evidence that protection against liver-stage malaria requires a combination of several antigens	Proteomics	Microarrays containing 23% the proteome were used to probe plasma from subjects with sterile protection or not induced by RAS	19	*P. falciparum* clone 3D7	Sporozoite	(41)
2015	Characterization of antigens involved on RAS protective immunity	Bioinformatics	Bioinformatic analysis, protein expression using wheat germ cell-free system, and validation in RAS-immunized volunteers	21	*P. falciparum* clone 3D7	Sporozoite	(42)
2016	Identification of *P. falciparum* highly expressed genes and validation in murine vaccines models	Transcriptomics and vaccination	Tiling microarray of *P. falciparum* liver stage vs. sporozoite or blood stage and production of ortholog vaccines in *P. yoelii* and *P. berghei*	124 genes upregulated in liver-stage, 6 protective antigens	*P. falciparum* isolate NF54	Liver-stage	(43, 44)

*N/A, Not applicable*.

#### Antigen Identification Based on Clinical and Challenge Studies

Remarkably, sterile immunity to a challenge with infectious sporozoites can be obtained through immunization with RAS (10, 45), through genetically attenuated parasites (GAP) (46, 47), and through chemoprophylaxis with infectious sporozoites (CPS) (48, 49), both in animals and humans. The protection induced occurs in the pre-erythrocytic stages (11), and is mediated by CD8^+^ T cells (50). Benefiting from this fact and from the genome and proteome studies of 2002, Doolan has identified 16 pre-erythrocytic antigenic proteins recognized by volunteers immunized with radiation-attenuated *P. falciparum* sporozoites, using bioinformatics predictions, HLA analysis, and *in vitro* cellular assays (16).

Also, benefitting from the genome sequencing, transcriptomic studies were performed in an attempt to identify new pre-erythrocytic antigens. By using suppression subtractive hybridization (SSH) of *P. yoelii* sporozoites and comparing them to merozoites, Kappe has identified 25 genes expressed in pre-erythrocytic stage, including the well-known CSP and TRAP (35). Although some interesting antigens were identified, this study may have excluded many antigens that are co-expressed in both sporozoite and merozoite stages.

In an attempt to characterize the transcriptome of liver-stage malaria, axenically cultured EEFs of *P. yoelii* were produced, which resulted in the identification of 652 unique transcripts based on 1,453 expressed sequence tags from cultured EEFs (36). Even though a number of transcripts were identified, axenic cultures do not mimic the natural hepatocyte infection, thus making this system poorly suited for the identification of liver-stage expressed antigens. To look into *in vivo* expression of parasite mRNA during liver-stage, Aguiar used laser capture microdissection to produce enriched samples of parasite mRNA for the construction of a liver-stage cDNA library, resulting in the expression of 623 unique *P. yoelii* genes (37).

Dominique Mazier hypothesized that *P. falciparum* sporozoites would undergo changes in gene expression during the transition from the insect to the mammalian host, in order to be prepared for the liver-stage. *P. falciparum* sporozoites were co-cultured *in vitro* with primary human hepatocytes and maintained at 37°C, to mimic this transition, which resulted in the identification of 532 up-regulated genes, suggesting that the salivary gland sporozoites are indeed activated for hepatocyte invasion upon contact with these cells at 37°C (39). Many interesting antigens were identified in this study, and later evaluated as pre-erythrocytic vaccine candidates (51).

In another study, RNA was collected from *P. yoelii* wild type, RAS and of developmental liver-stage samples obtained by laser microdissection at 24 and 48 h post-infection. Transcriptional analysis made with microarrays on these samples identified 1,133 genes significantly differentially expressed compared to blood stages (40).

Doolan et al. have produced microarrays containing 23% of *P. falciparum* proteome and used them to probe plasma from subjects with sterile protection or no protection after experimental immunization with RAS. Nineteen pre-erythrocytic stage antigens were strongly associated with sporozoite-induced protective immunity, 16 of which were novel. This study revealed that sterile protection against malaria requires a combination of several antigens, and the authors suggested that a malaria vaccine should be multivalent in order to improve its efficacy (41).

Another study based on bioinformatic analysis and expression databases produced a list of 27 recombinant proteins using wheat germ cell-free protein expression system. Twenty-one proteins were recognized by plasma and 20 by PBMCs from RAS-immunized volunteers (42).

Using tiling microarrays, Duffy and colleagues identified 124 *P. falciparum* genes expressed in liver-stage compared to sporozoite or blood-stage. Produced 21 of vaccines with orthologs in *P. yoelii* and *P. berghei*, six being protective and two offered improved protection when in combination with CSP, compared to CSP alone (43, 44). Many other studies followed these with the purpose of identifying liver-stage antigens, to be used as vaccines or drug targets and to understand the pathophysiology of liver-stage malaria and host-pathogen interactions.

### Liver-Stage Antigens

Sterile protection against malaria can be obtained through immunization with RAS. Based on this observation, many studies were performed to identify antigens expressed during liver-stage that would be associated with protection. CSP was the first antigen to be described conferring protection at the pre-erythrocytic level. CSP is predominantly expressed in the sporozoite and early liver forms (52). Due to incomplete protection provided by CSP, comparing to immunizations with radiation-attenuated sporozoites, several studies attempted to identify other antigens expressed at this stage, resulting in the identification of LSA-1 (29), STARP (31), SALSA (32), LSA-3 (33) and others recently described in a comprehensive review (53).

The first pre-erythrocytic antigen identified was the circumsporozoite protein (CSP), in 1980 (Tables 1, 2). An hybridoma resulting from the fusion of myeloma cells with splenocytes from mice immunized with *P. berghei* was generated and its antibodies recognized the surface of *P. berghei* sporozoites (28) (Table 2). RTS,S/AS01 is a recombinant protein vaccine containing part of the CSP antigen, including 19 NANP repeats and the carboxyl terminus, expressed in virus-like particle hepatitis B surface antigen and formulated with AS01 adjuvant (3, 59).

**Table 2:** Pre-erythrocytic antigens used in vaccines.

**Antigen**					**Vaccine**		**References**
**Name**	**Symbol**	**Size (aa)**	Accession^*^	**Expression**	**Name**	**Phase**	
Circumsporozoite protein	CSP	397	PF3D7_0304600	Sporozoite and liver-stage	RTS,S/ASO1	Phase III	(3, 54)
Thrombospondin-related anonymous protein	TRAP	574	PF3D7_1335900	Liver-stage and gametocytes	ChAd63	Phase IIb	(30, 54, 55)
Liver stage antigen 1	LSA1	1,162	PF3D7_1036400	Liver-stage	ChAd63 and MVA	Pre-clinical	(29, 51, 54, 56)
Liver stage antigen 3	LSA3	1,558	PF3D7_0220000	Liver-stage and blood stage	ChAd63 and MVA	Pre-clinical	(51)
Liver stage associated protein 1	LSAP1	106	PF3D7_1201300	Sporozoite and liver-stage	ChAd63 and MVA	Pre-clinical	(51)
Liver stage associated protein 2	LSAP2	302	PF3D7_0202100	Sporozoites, liver-stage and early ring stage	ChAd63 and MVA	Pre-clinical	(51)
Early transcribed membrane protein 5	ETRAMP5	181	PF3D7_0532100	Sporozoite, liver-stage, blood-stage	ChAd63 and MVA	Pre-clinical	(51)
Cell traversal protein for ookinetes and sporozoites	CelTOS	182	PF3D7_1216600	Sporozoite, liver-stage and gametocytes	ChAd63 and MVA	Pre-clinical	(51, 57)
Protein UIS3	UIS3	229	PF3D7_1302200	Sporozoite, liver-stage and blood-stage	ChAd63 and MVA	Pre-clinical	(51)
Falstatin	Falstatin	413	PF3D7_0911900	Sporozoite, liver-stage and blood-stage	ChAd63 and MVA	Pre-clinical	(51)
Liver specific protein 1	LISP1	3,597	PF3D7_1418100	Liver stage	DNA	Pre-clinical	(43, 44)
Sporozoite and liver stage asparagine-rich protein	SLARP	2,940	PF3D7_1147000	Liver stage	DNA	Pre-clinical	(43, 44)

* *P. falciparum clone 3D7 accession numbers obtained from PlasmoDB Release 38 (58)*.

The first liver-stage antigen identified was the Liver Stage-Specific-Antigen-1 (LSA-1). The authors searched for sera from patients that were restricted to pre-erythrocytic stages. One such individual, living in a malaria-endemic area and undergoing continuous drug prophylaxis for 26 years, had high antibody titers against sporozoites and liver-stage, yet negative for blood-stage. Serum was used to screen against a clinical strain *P. falciparum* genomic expression library, which led to the identification of clones expressing LSA-1. One clone was sequenced and contained a DNA fragment of 196 bp composed of a 51 bp repeat sequence, encoding the 17 amino-acid sequence (EQQSDLEQERLAKEKLQ) recognized in ELISA by affinity-purified human antibodies (29) (Table 2). The pre-erythrocytic antigens LSA3, LSAP1, LSAP2, ETRAMP5, UIS3, and Falstatin, used in pre-clinical and clinical vaccine development were identified in the abovementioned Mazier's sporozoite screen (Table 2).

## Tools to Study Liver-Stage Malaria Biology

Although many attempts to characterize liver-stage proteins have been performed in the last 50 years, the difficulties in performing these studies stem from three main reasons: first, it is difficult, laborious and complex to obtain large numbers of *P. falciparum* sporozoites required for hepatocyte infection experiments; secondly, primary hepatocytes are poorly suited for research because they show great phenotypic variability across donors and hepatoma cell lines are not sufficiently metabolically mature when compared to primary human hepatocytes; thirdly, the sporozoite infectivity, i.e., the percentage of infected hepatocytes, is traditionally very low. This will be discussed in greater detail below.

### Parasites

During transmission, sporozoites are injected with mosquito saliva into the skin. The current methods for obtaining viable sporozoites for liver stage studies include the well-known hand dissection of mosquitoes, followed by grinding the salivary glands with a pestle to release sporozoites (60), passing the glands through a needle and syringe (61), or purifying the sporozoites through a density gradient (62), which may produce a heterogeneous mix of sporozoites, mosquito debris, salivary glands, and mosquito saliva. Indeed, an infected *Anopheles* mosquito saliva protein that is associated with saliva sporozoites was recently identified by mass spectrometry, with similarity to the human gamma interferon inducible thiol reductase (GILT), and has a negative impact on the speed and cell traversal activity of Plasmodium (63).

Animal models have been used for more than half a century to ascertain the efficacy (10) and immunogenicity (64) of vaccines against malaria. However, malaria mouse models do not allow the successful infection of the human parasite *P. falciparum*. The rodent malaria parasites (RMP) *P. yoelii, P. berghei* and *P. chabaudi*, have been extensively used as models for the human disease. RMPs have more than 90% homology with primate parasites such as *P. falciparum* (65). However, some *P. falciparum* vaccine candidates don't have orthologs in the murine malaria parasites, as is the case of LSA1 (29), some antigens are present in both but not with complete overlapping sequences, as is the case of CSP (30), and their expression might be species specific, as is often the case with many genes (66). Furthermore, it has been suggested that transcriptional evolution in *Plasmodium* species may be under different selection pressures, due to host specific variations (66). To overcome this, transgenic parasites have been developed to express *P. falciparum* antigens, so that these antigens can be assessed for immunogenicity in murine models. Moreover, transgenic RPMs that express unique *P. falciparum* antigens can be used to assess the efficacy of vaccines containing the same antigens, in mice. For a recent review on this subject please refer to Longley et al. (67).

RMPs expressing *P. falciparum* genes can be produced by genetic manipulation based on the insertion of exogenous DNA through homologous recombination mechanisms and transfection methods (68). *P. falciparum* genes can be either introduced to replace a given RMP endogenous gene or to introduce an additional gene (67). Recently, CRISPR/Cas9 technology has emerged as an improvement in the generation of transgenic parasites (69).

New tools to study pre-erythrocytic malaria are urgently needed. These include *Plasmodium* parasites expressing reporter molecules throughout the entire life cycle, but especially detectable in the liver-stage. Reporter molecules sensitive to changes in the pH, oxidative stress or other intracellular conditions would be of particular interest to pinpoint metabolic changes in the parasitized cell, which could provide additional information on the host-pathogen interactions during infection. Of particular interest is the recent *P. berghei* reporter parasite line that reveals membrane dynamics by GFP-tagging of a non-essential protein localized in the plasma membrane, and its trafficking in living parasites through the entire life, cycle using live-cell microscopy can be followed (70).

Recently, new *P. falciparum* clinical isolates NF135 and NF166 were identified by Sauerwein, that present a significantly higher infectivity on human primary hepatocytes *in vitro*, around 3%, and showed faster egress to the blood compared to NF54, which correlated directly with the magnitude of the first wave of blood-stage parasites to emerge from the liver *in vivo*, and correlated inversely with the pre-patent period in controlled human malaria infection (CHMI) subjects (71).

Even though some progress has been achieved by the introduction of reporter genes, development of transgenic strains and characterization of new clinical isolates, the difficulties of producing pre-erythrocytic forms in high numbers, hinders the identification of liver-stage antigens.

### Hepatocytes

The liver, the largest human organ, is the target of the sporozoites upon invasion. At the cellular level, the architecture of the liver is organized in hexagonal lobules composed of several cell types from the most abundant hepatocytes, to endothelial cells, cholangiocytes, stellate cells, küppfer cells, dendritic cells and resident lymphocytes. These are supplied by an intricate network of bile canaliculi and sinusoids, leading to bile ducts and central and portal branch veins, respectively, which in turn, combined with the hepatic branch artery, constitute the portal triad. This highly organized organ, with a complex three-dimensional architecture, is involved in crucial functions of metabolism, storage, and detoxification as well as endocrine and exocrine functions (72–74).

Early studies in pre-erythrocytic malaria were performed in microscopic observations of liver sections (75), and later in monolayers of hepatoma cell lines (76). Currently, primary cells from human, non-human primate, murine and rabbit cells have been extensively used, as well as hepatoma cell lines (77). More recently, induced pluripotent stem cells-derived hepatocyte-like cells (iHLCs), have been shown to support pre-erythrocytic malaria using *P. berghei, P. yoelii, P. falciparum*, and *P. vivax* (78). This variety of cultivable cell types promoted a rapid improvement in the understanding of the biology of EEFs. However, primary hepatocytes fail to proliferate *in vitro* in contrast to their natural regenerative ability *in vivo*. Furthermore, although primary hepatocytes are metabolically competent, they are phenotypically unstable, exhibit great variability between batches and possess limited ability to proliferate *ex vivo* (79–81). In contrast, hepatoma cell lines proliferate generously, but lack several maturity markers, with especial relevance to a reduced Cytochrome P450 gene expression (79, 80), making them poorly suited for the complete understanding of liver-stage malaria infection. Several hepatoma cell lines are commercially available both from murine or primate origin. For a review on this subject please read the useful Prudêncio' s toolbox article describing all host-parasite combinations of Plasmodium hepatic infection models, *in vivo, ex vivo*, and *in vitro* (77).

Models of two-dimensional (2D) monolayer cultures in a supportive matrix (76, 82, 83) or 2D co-cultures of primary hepatocytes surrounded by stromal cells (61), have been used to study pre-erythrocytic malaria. The latter were improvements on the monolayers, but still lack the three-dimensional architecture of an organ. To overcome that, 3D models have been developed, containing several cell types organized in a three-dimensional structure. A further improvement, these co-cultured cells were organized in a functional matrix to mimic the organ, known as organoids. Although mimicking somehow the three-dimensional architecture of the organ, are not able to reproduce the physical dynamics of the blood and duct vessels and shear stress typical of the liver. To include the complex fluid dynamics in these models, new models of liver-on-a-chip engineered liver platforms have been designed to mimic the hepatic fluid dynamics environment in the liver (72). These models were originally developed with the purpose of drug testing and cell differentiation studies may be now employed for pre-erythrocytic malaria studies.

To reduce the gap between *in vitro* liver-mimicking models and *in vivo* human livers, humanized mice were developed. These are immunodeficient mice xenographed with human primary hepatocytes and constitute the closest *in vivo* models of human liver developed so far and were received both with enthusiasm and frustration.

### Mouse Models

Mice have been instrumental in our understanding of malaria pathogenesis and have driven most of the progress made in the field. The initial studies using mouse models were on inbred A/J mice (10, 11, 84, 85), BALB/c (28, 86), C57BL/6 (87, 88), as well as outbred Theiler's Original (T.O.) mice (89). Experiments in mice revealed that CD8^+^ T cells are required for protection against a RAS challenge, while CD4^+^ T cells are dispensable, by depleting CD8^+^ and CD4^+^ T cells respectively, in immunocompetent mice (84). Additionally, IFN-γ and antibodies were also shown to be required for inhibition of the development of EEFs during protection induced by vaccination with RAS (84).

Naturally immunocompromised mice, as well as laboratory produced knock-out and transgenic mouse models have been used to improve our understanding of the disease. Congenitally athymic mice were used to demonstrate the T cell requirement for protection induced with RAS against a lethal challenge of *P. berghei* sporozoites (85). Beta 2-microglobulin knockout mice were instrumental in showing the requirement of MHC-I presentation to CD8^+^ T cells in inducing protection mediated by RAS (88). Transgenic mice were generated to express a T cell receptor specific for the epitope (SYVPSAEQI) from the circumsporozoite protein of *P. yoelii*. This peptide was recognized by transgenic CD8^+^ T cells but not CD4^+^ T cells, was able to inhibit parasite development (90) and its protection was independent of IFN-γ production (87). Even though a great diversity of mouse model exists, mice may not recapitulate the pathophysiology of human severe malaria, as in the case of human cerebral malaria (91). Therefore better models of disease are required.

Humanized mouse models consist of chimeric mice containing human cells, and have been developed in an attempt to provide *in vivo* animal models to study human disease. The first liver humanized mouse developed was based on a strain of immunocompromised SCID mice homozygous for the Alb-uPA transgene, which causes liver injury. The Alb-uPA transgenic mouse has an accelerated hepatocyte death and, as this strain is immunocompromised, human hepatocytes were successfully transplanted and repopulated the mouse liver (92). Moreover, these animals were successfully infected with hepatitis C virus (92), and later with *P. falciparum* (93).

Another model, the FRG mouse that lacks the fumarylacetoacetate hydrolase (Fah^−/−^) which causes liver injury, and immunocompromised (with a Rag2^−/−^ and Il2rγ^−/−^ background), can harbor up to 90% of human hepatocytes in the liver (94), and when crossed with NOD mice (non-obese diabetic mice that tolerate human hematopoietic cells), and transplanted with human hepatocytes and O^+^ human red blood cells, supports the transition of liver-stage to blood-stage malaria, after *P. falciparum* infection (95).

Recently a double engraftment of TK-NOG mice by human primary hepatocytes and red blood cells was developed (96). For the first time, this mouse model allows the complete hepatic development of *P. falciparum*, and the transition to erythrocytic stages, including the appearance of mature gametocytes. Even though the human hepatocytes that repopulate the mouse liver account for only 60–80% of total hepatocytes, this mouse model closely mimics the physiological complexity and specificity of an *in vivo* infection in the human environment.

Another recent model consists in engineered artificial human livers, implantable in mice without the requirement of liver injury. Composed of macroporous PEG cryogels, the human ectopic artificial liver (HEAL) is amenable to liver stage *Plasmodium* infection *in vitro* and *in vivo*. With intraperitoneal implantation and support infection with both liver stage rodent and human *Plasmodium* parasites *in vivo* (97).

Although these mouse models are attractive and offer the possibility of studying pre-erythrocytic malaria *in vivo* with *P. falciparum* and other human pathogenic *Plasmodium* parasites, these animals are immunodeficient thus not allowing the study of vaccine efficacy and immunogenicity, due to a constitutive lack of immune response.

Another humanized mouse model, the DRAG (HLA-DR4, Rag1^−/−^, Il2rγ^−/−^, NOD) mice, were generated by transplantation of HLA-II-matched human Hematopoietic stem cells (HSC) and reconstituted human hepatocytes, küpffer cells, liver endothelial cells, and erythrocytes, allowed the full vertebrate life cycle of *P. falciparum* and developed functional human T and B cells (98).

Even though none of these mouse models has served to identify antigens expressed by the liver stages of *P. falciparum*, they have been instrumental to study the dynamics of host-parasite interactions, to dissect the immune response against RMPs and vaccine efficacy and immunogenicity evaluation. For a recent review liver-stage response against malaria antigens using mouse models, please see (99).

### Infectivity

Infectivity, or rate of infection of sporozoites in hepatocytes, can be determined semi-quantitatively by fluorescence microscopy, quantitatively by flow cytometry, and indirectly by RT-PCR. For both fluorescence microscopy and flow cytometry, the parasite has to be labeled with a fluorophore to be detected. For RT-PCR, a standard curve with a known copy number of Plasmodium genomes has to be produced, which serves as a ruler to measure the amount of parasites in a given sample. Then, if the total number of hepatocytes is known, a rate of infection can be estimated by RT-PCR. Usually, these three methods correlate relatively well. It has been reported that <5% cells from hepatoma cell lines are infected with murine parasites in most publications (100, 101), however, infectivity's of nearly 10% could be reached when small numbers of hepatocytes were infected with *P. berghei* (86). In several reports, <2.5% of hepatoma cell lines were infected with *P. falciparum* (61, 102). In frozen human primary hepatocytes, 0.2% or less of cells were infected with murine parasites (103) and lower than 0.3% were infected with *P. falciparum* (61). In freshly harvested human primary hepatocytes, between 1 and 3% of cells were infected with *P. falciparum* (71), in contrast to 0.2% when infected with RMPs. Primary mouse hepatocytes and mouse cell lines, have had infectivity's lower than 6 and 2%, respectively, using RMPs, based on immunofluorescence microscopy or flow cytometry (Table 3). The lower infectivity's observed are a typical feature of malaria liver-stage infection and depend not only on the origin of the host cells and strain of parasite, but also on the expression of CD81 receptor, which has been described as essential for primary human hepatocyte invasion (107, 109). The infectivity may vary due to the expression levels of EhpA2 (110) and the class B, type I scavenger receptor (SR-BI) (111). Additionally, primary hepatocytes in humanized mouse models, as well as obtained from genetic altered mice may display different levels of infectivity. Primary hepatocytes originating from other animal models such as old and new world monkeys, rabbits and rats, may offer different infectivity's *in vitro*. Also, the treatment of hepatocytes with diverse compounds may change the rate of infection. Interestingly, it has been reported a staggering 10–20% rate of infection in irradiated HepG2 cells infected with *P. berghei* (89). Large scale antigen discovery experiments using current methods depend on large number of infected cells, therefore, it is extremely laborious and time-consuming to obtain sufficient number infected hepatocytes *in vivo, ex vivo*, and *in vitro*. Thus, the identification of new antigens has been hindered.

**Table 3:** The malaria parasite rate of infection in hepatocytes.

	**Human hepatocytes**			**Mouse hepatocytes**	
	**Hepatoma^a^**	**Primary**		**Cell lines^b^**	**Primary**
		**Frozen**	**Fresh**		
Murine malaria parasite(*P. berghei* or *P. yoelii*)	<10% (86, 101, 104–106)	<0.2% (103)	<0.2% (104)	<6% (100, 105, 106)	<2% (105)
Human malaria parasite(*P. falciparum* NF54or 3D7, or *P. vivax*)	<2.5% (61, 83, 102, 107, 108)	<0.3% (61, 107)	1–3% (71)	N/D	N/D

a *Cell lines include HC04, Hep G2, Huh7, WI38*.

b *Cell lines include Hepa1-6 and Bnl-1Me A.7R.1*.

*N/D, Not described*.

## Antigen Identification for Vaccine Development

On the pre-genomic era, vaccine candidate identification was relatively empiric. Surprisingly, most of the current vaccine candidates were identified before the publication of *P. falciparum* genome, and they were mostly based on the screening of cDNA libraries for functional antibodies (112). The post-genomic era, initiated with the publication of *P. falciparum* genome (26), followed by the first transcriptomics (113, 114) and proteomic studies (27, 34), alone or in combination (38, 115), provided the tools for the systematic interrogation of the biology of the parasite at every stage. A consequence of the genome sequencing, allowed the combining data from genomics, transcriptomics and proteomics studies, with an unprecedented depth allowing the identification of new vaccine candidates, promoting the exponential creation and development of new bioinformatics tools, with or without experimental validation, opening new avenues for the discovery and functional analysis of new vaccine antigens at a faster pace and larger scale (116). This led to development of a more rational approach to identify vaccine candidates termed reverse vaccinology.

### Reverse Vaccinology

Reverse vaccinology consists of the integration of whole genome sequencing data from a pathogen and the identification of vaccine candidates using bioinformatics as was employed for the first time by Rappuoli et al. on a virulent strain of *Neisseria meningitidis* serogroup B. Bioinformatics analysis were performed to shortlist 350 candidate antigens, followed by expression and purification of selected antigens in *E. coli*, and its immunization in mice. Sera from immunized mice were used to identify seven antigens that were positive all three assays: ELISA against whole cell MenB, FACS to detect proteins at the surface of MenB and MenB bactericidal activity (117). This strategy allows for the reduction of number of candidate antigens and has been extensively applied on number of subsequent studies identifying vaccine antigens against group B streptococcus (118), *Chlamydia pneumoniae* (119), Streptococcus pneumoniae (120), *Bacillus anthracis* (121), *Porphyromonas gingivalis* (122), among other bacterial pathogens with relatively small genomes. Reverse vaccinology in parasitology, and more specifically in malaria has been relatively successful in identifying some potential antigens for vaccine development. For example, Mu et al. searched for polymorphisms in ~65% of *P. falciparum* genes and identified several polymorphic loci. From a list of 56 antigens, half of those being already known, some were confirmed as potential vaccine candidates, using human immune sera (123), one of which the Apical membrane antigen 1 (AMA1), a well-known blood-stage vaccine candidate (124). Reverse vaccinology has been applied to malaria, particularly in the identification of the transmission blocking vaccine candidates, discussed in a recent review (125).

### New Challenges in Antigen Identification—Immunopeptidomics

Large scale proteomic studies on parasite forms are a rapid and sensitive manner to discover new vaccine candidates. However, from the 5,300 predicted proteins and 3,000 described so far, the identification of the most immunogenic antigens is not a trivial task, not only due to the large number of potential candidates but also because strong immune responses may not correlate with protection. During liver stage malaria, infected hepatocytes present pathogen peptides through MHC Class I molecules from some of the many hundreds to thousands of genes expressed by the liver-stage parasite. Which genes are most abundantly presented as peptides on HLA class I molecules on the surface of hepatocytes that can be targeted by protective CD8^+^ T cells, is still largely unknown.

The peptides presented by MHC molecules are designated as the immunopeptidome (126). MHC-I derived peptides are typically 9–12 amino acids long and, based on the current methods, the minimal requirements for the identification of MHC-I derived peptides using mass spectrometry requires a sample containing 5 × 10^8^ cells expressing ~2 × 10^5^ MHC molecules per cell (126). Additionally, for the identification of Plasmodium peptides in a sample containing infected cells, the requirements may be more stringent as *P. falciparum* and *P. berghei* may interfere with MHC-I antigen presentation at the late time-points of liver-stage (104).

In the proteomics studies presented on Table 1, tryptic peptides were produced for the mass spectrometry analysis. Trypsin cleaves peptides at the C-terminal lysine and arginine residues (127). Enquiring peptides with Lys or Arg residues at the C terminus, although facilitates the identification of proteins, the majority of tryptic peptides (56%) are too small (≤6 residues) and thus not identified by mass spectrometry (127). An important advantage of immunopeptidomics is that no digestion protocol is used on the pathogen peptides, thus the identification of non-tryptic peptides that is, of native peptides (≥7 residues), can be very challenging although possible, however with a relatively low identification success rate of 10% compared to 50% for trypsinized peptides (126).

Moreover, as the rate of infection of hepatocytes infected with *Plasmodium* spp. is extremely low, arguably 5% (see Table 3), the mass spectrometry identification of pathogen peptides would require a higher number in the order of 1 × 10^10^ infected hepatocytes at a 5% rate of infection, which makes extremely laborious and complex to produce samples this size.

There is hope as mass spectrometry has been successful in the identification of MHC-II derived peptides from *Leishmania major*, from infected bone marrow-derived dendritic cells. Synthetic peptides were able to activate CD4^+^ T cells, and vaccination with the main synthetic peptide from Glycosomal phosphoenolpyruvate carboxykinase (PEPCK) protein, recombinant protein or DNA vaccines induced protection in mice. Although the protection was relatively long and protected from a *L. donovani* challenge as well, sterile protection was not achieved (128). More recently, a survey on the MHC-II immunopeptidome was conducted on murine blood-stage malaria, generating a list of 14 MHC-II ligands presented by cDC1 dendritic cells, which included the blood-stage vaccine candidates AMA1 and MSP1 (129).

The identification of antigens as vaccine candidates against malaria based on immunopeptidomics is in its infancy and major achievements should be produced in the upcoming years.

## Discussion

The development of a useful malaria vaccine is a major priority for tropical infectious diseases: mortality from malaria exceeds half a million each year and there are hundreds of millions of cases. Control is threatened by increasing drug and insecticide resistance. A highly effective malaria vaccine could be extremely valuable for disease control but this has been very difficult to develop. However, there are increasing signs that success is possible and maybe even in sight. RTS,S/AS01, a sporozoite vaccine with modest efficacy may be licensed in the next 5 years. A liver-stage vaccine recently showed 67% efficacy in preventing malaria infection in Kenyan adults (55) and intravenous cryopreserved sporozoites can provide high level efficacy in challenge studies (130).

However, a cost-effective vaccine with high level durable efficacy in young infants remains elusive. The genome of *P. falciparum* is yielding new antigens that show promise such as PfRH5 (131) at the blood-stage, PfLSA1 (51), PfLSAP2 (51) and PfCelTOS (51, 57) at the pre-erythrocytic stage. New antigens are urgently needed to achieve the highest possible protection efficacy against malaria. Most likely, a combination of antigens expressed in every stage of the parasite's life cycle would improve the efficacy of a malaria vaccine.

Viral vector vaccines have the capacity to induce strong protective T cell responses against pathogens (25). For more than 20 years our group has been developing vaccine strategies with Adenovirus and MVA as antigens delivery strategies for vaccination, both in pre-clinical and clinical trials, with more than 1,500 people vaccinated in several countries. This viral vector vaccine approach, has been extensively reported to be safe, well-tolerated and relatively easy to deploy in remote settings (132). Viral vectored vaccines expressing new liver-stage antigens, alone or in combination, are expected to provide new and exciting antigens in the near future.

The identification of new liver-stage sub-unit vaccine candidates against malaria requires both the exploration of the genomics, transcriptomics and proteomics tools already developed, as well as new tools, based in three main pillars.

First, sporozoites: parasite strains with higher infectivity, such as NF135 and NF166 that improve the rate of infection of hepatocytes (71), may have promising consequences for the field. New genetic manipulation tools could facilitate the generation of diverse genetic attenuated strains or transgenic strains able to express multiple antigens simultaneously under different promoters. A good example was the generation of a resource developed by Billiker and colleagues that consists in a large-scale reverse genetic screening method based on barcoded vectors inserted into the *P. berghei* genome, which can be measured through barcode sequencing. This resource can provide the identification of essential genes involved in the parasite development. Additionally, this allows the study of the function of each barcoded KO genes, phenotyping each of these mutants, which can provide in depth information on the function of each gene, as the authors showed by studying the function of a set of promiscuous kinases (133). Improved methods to dissect and obtain pure sporozoites should also be developed to promote better yields of infected hepatocytes. Alternatively, improvements in the development of axenic cultures could offer an abundant source of sporozoites.

Second, hepatocytes: improved methods for culturing primary hepatocytes as well as the generation of fully matured and differentiated hepatocytes derived from stem cells (79), would be advantageous for the improvement of liver-stage infection experiments, especially to reduce the extensive variability observed as a consequence of the diversity of primary cells. Further progress, taking advantage of stem cell models for infection in combination with humanized mice or 3D organoids, could provide new models for infection. Humanized mice are the first *in vivo* model of a human liver, reason for great excitement in the field, but these mice are hard to maintain, and suffer from the inherent genetic variability of the primary human hepatocytes, which limit large-scale experiments. Improvements in the scalability and engraftment of these models could produce models with several humanized tissues to further explore the interaction between the liver, blood and with other tissues as well. Additionally, the development of new delivery methods of sub-unit vaccines specifically designed to target the liver could provide more robust protection and a better understanding of the protection mechanisms at the site of infection, *in vivo*.

Third, antigen identification: although there is currently a fair number of transcripts and proteins known to be expressed in the liver-stage, not all highly expressed proteins might be presented by MHC-I molecules, hence, not being recognized by CD8^+^ T cells. The pathogen peptides that are processed and presented via MHC-I, by the infected hepatocytes are largely unknown. Thus, identifying these peptides can provide and prioritize new vaccine candidates. To identify pathogen-specific MHC-associated peptides, a large number of infected cells are required, which depend on three key aspects: (i) the difficulty in obtaining large number of infectious sporozoites, (ii) the difficulty in obtaining a reliable source of human hepatocytes and (iii) the low yield of infection, or low infectivity. A combination of improvements in these three key aspects, with the current methods for antigen identification by mass spectrometry, would offer new vaccine candidates.

Other promising strategies for identifying liver-stage antigens have been described. Among these, bioinformatic analysis of MHC binding predictions to identify and select T cell epitopes as new antigen vaccine candidates (134). Also, *in silico* approaches, consisting of variations of reverse vaccinology combined with immunological analysis. Based on samples collected in CHMI or natural exposure to malaria, large-scale screens have been performed to identify targets able to induce T cell or antibody responses (135).

Although significant progress has been achieved in the last 50 years, culminating with the implementation of the only malaria vaccine in Phase III clinical trials RTS,S/AS01, exciting progress is expected to happen in the coming years, to improve the efficacy of current vaccines toward the final objective of malaria eradication.

## Author Contributions

PB conceived and wrote the article.

### Conflict of Interest

The author declares that the research was conducted in the absence of any commercial or financial relationships that could be construed as a potential conflict of interest.
